# Feasibility and tolerability of reduced-volume oral sulfate solution combined with elobixibat for bowel preparation before colonoscopy: a single-center pilot study

**DOI:** 10.1093/gastro/goag063

**Published:** 2026-07-07

**Authors:** Takashi Murakami, Kentaro Ito, Eiji Kamba, Naoki Tsugawa, Yuka Hirasawa, Wataru Hata, Yudai Otsuki, Tomonori Yamauchi, Tomoyoshi Shibuya, Akihito Nagahara

**Affiliations:** Department of Gastroenterology, Juntendo University Faculty of Medicine, Tokyo 113-8421, Japan; Department of Gastroenterology, Juntendo University Faculty of Medicine, Tokyo 113-8421, Japan; Department of Gastroenterology, Juntendo University Faculty of Medicine, Tokyo 113-8421, Japan; Department of Gastroenterology, Juntendo University Faculty of Medicine, Tokyo 113-8421, Japan; Department of Gastroenterology, Juntendo University Faculty of Medicine, Tokyo 113-8421, Japan; Department of Gastroenterology, Juntendo University Faculty of Medicine, Tokyo 113-8421, Japan; Department of Gastroenterology, Juntendo University Faculty of Medicine, Tokyo 113-8421, Japan; Department of Gastroenterology, Juntendo University Faculty of Medicine, Tokyo 113-8421, Japan; Department of Gastroenterology, Juntendo University Koshigaya Hospital, Saitama 343-0032, Japan; Department of Pathophysiological Research and Therapeutics for Gastrointestinal Diseases, Juntendo University Faculty of Medicine, Tokyo 113-8421, Japan; Department of Gastroenterology, Juntendo University Faculty of Medicine, Tokyo 113-8421, Japan; Department of Pathophysiological Research and Therapeutics for Gastrointestinal Diseases, Juntendo University Faculty of Medicine, Tokyo 113-8421, Japan

**Keywords:** bowel preparation, oral sulfate solution, elobixibat, reduced-volume regimen, feasibility study

## Abstract

**Background:**

Reducing the volume of bowel preparation may improve patient adherence and tolerability without compromising cleansing efficacy. Oral sulfate solution (OSS) is a low-volume preparation option, and elobixibat, a bile acid transporter inhibitor, may enhance colonic secretion and motility. We evaluated the feasibility, efficacy, safety, and tolerability of a same-day regimen combining elobixibat with OSS.

**Methods:**

This prospective single-arm pilot study was conducted at Juntendo University Hospital in Japan. Consecutive patients aged 20–64 years scheduled for elective colonoscopy were enrolled between April 2021 and March 2023. Patients received elobixibat (10 mg once daily for 3 days before colonoscopy), followed by same-day OSS using a “stop-when-clear” protocol. The primary outcome was adequate bowel preparation, defined as a total Boston Bowel Preparation Scale (BBPS) score ≥6. Secondary outcomes included OSS volume consumed, adverse events, and patient-reported tolerability assessed by questionnaire.

**Results:**

Twenty-eight patients were analyzed. Adequate bowel preparation was achieved in 96% of patients. BBPS ≥2 were 82% in the right colon, 96% in the transverse colon, and 100% in the left colon. The mean total OSS volume was 771 mL, and 57% of patients completed preparation with ≤720 mL. No serious adverse events were observed. Most patients reported acceptable palatability (82%) and manageable volume (100%). Among those with prior colonoscopy experience, 75% preferred this regimen and 86% indicated willingness to use it again.

**Conclusions:**

A same-day regimen combining elobixibat with OSS demonstrated favorable feasibility, efficacy, and tolerability. Reduced OSS volume may be achievable in selected patients. However, these findings are limited to a relatively low-risk, non-elderly population and should be confirmed in larger comparative studies.

## Introduction

Adequate bowel preparation is a prerequisite for high-quality colonoscopy, as insufficient cleansing is associated with prolonged procedure time, missed neoplastic lesions, and the need for repeat examinations [1, 2]. Current guidelines emphasize the importance of achieving adequate bowel cleansing to ensure diagnostic accuracy and therapeutic safety [2].

Polyethylene glycol (PEG)-based regimens have been widely used as standard bowel preparation; however, they typically require ingestion of large volumes of solution (up to 2–4 L), which may impair patient compliance and tolerability [3]. High-volume intake is frequently reported as one of the most burdensome aspects of colonoscopy preparation and can lead to incomplete consumption, nausea, bloating, and reduced patient satisfaction [4]. Therefore, strategies to reduce preparation volume while maintaining adequate cleansing efficacy remain an important clinical objective.

Oral sulfate solution (OSS) was developed as a lower-volume alternative and has demonstrated non-inferior cleansing efficacy compared with PEG-based regimens in randomized controlled trials [5, 6]. Despite the reduced volume, patient burden related to intake amount and gastrointestinal discomfort may still limit adherence in some individuals.

Elobixibat, an ileal bile acid transporter inhibitor, enhances colonic motility by increasing bile acid delivery to the colon and is approved for the treatment of chronic constipation [7, 8]. Given its pharmacological mechanism, elobixibat may augment bowel evacuation and potentially allow further reduction in preparation volume when combined with conventional agents. However, clinical data evaluating its role in colonoscopy preparation are limited [9].

We therefore conducted a single-center pilot study to evaluate the feasibility, cleansing efficacy, safety, and patient tolerability of a reduced-volume OSS regimen combined with elobixibat. The primary aim was to assess whether acceptable bowel cleansing could be achieved in real-world practice with reduced OSS intake.

## Materials and methods

### Study design and patients

**Figure 1:**
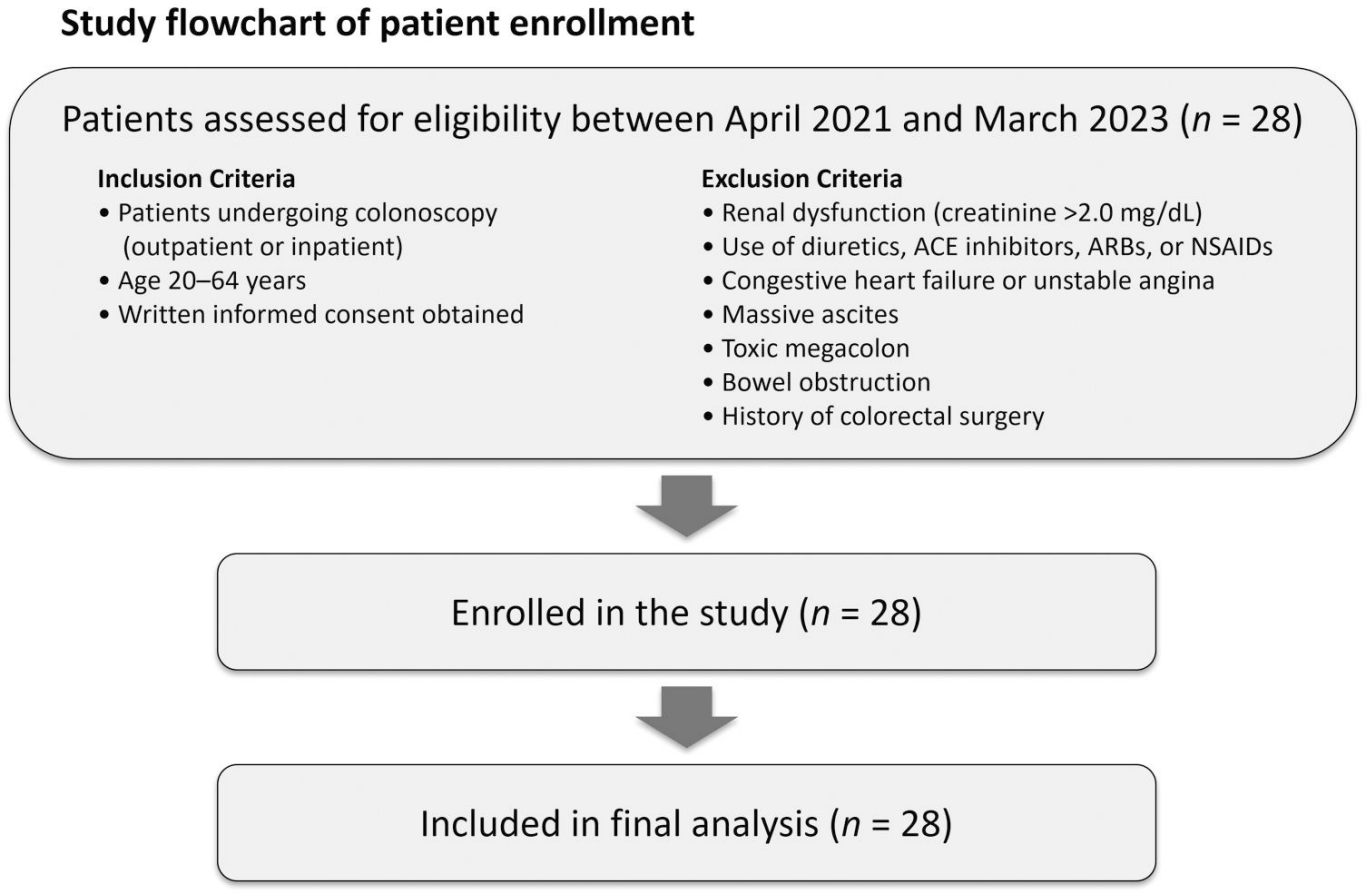
Inclusion and exclusion criteria for study enrollment. Patients undergoing colonoscopy were screened for eligibility. Inclusion criteria were age 20–64 years and provision of written informed consent. Exclusion criteria included age ≥65 or <20 years, renal dysfunction (creatinine >2.0 mg/dL), use of medications affecting renal function (diuretics, ACE inhibitors, ARBs, or NSAIDs), congestive heart failure, unstable angina, massive ascites, toxic megacolon, bowel obstruction, or prior colorectal surgery. Eligible patients were enrolled in the study and included in the final analysis.

This was a prospective single-arm pilot study conducted at Juntendo University Hospital (Tokyo, Japan) between April 2021 and March 2023. Consecutive patients scheduled for elective colonoscopy were screened for eligibility. The study protocol was approved by the Institutional Review Board and Ethics Committee of the Juntendo University Hospital (registration #E21-0151) and conducted in accordance with the Declaration of Helsinki. Written informed consent was obtained from all participants prior to enrollment. The detailed inclusion and exclusion criteria used for patient selection are summarized in Fig. 1.

Patients were eligible for inclusion if they met all of the following criteria:

- Patients requiring colonoscopy, regardless of outpatient or inpatient status and regardless of sex;
- Age between 20 and 64 years;
- Provision of written informed consent.

Patients were excluded if they met any of the following criteria:

- Acute or chronic renal dysfunction (serum creatinine >2.0 mg/dL);
- Current use of medications that may affect renal blood flow or renal function (diuretics, angiotensin-converting enzyme inhibitors, angiotensin II receptor blockers, or nonsteroidal anti-inflammatory drugs);
- Congestive heart failure or unstable angina;
- Massive ascites;
- Toxic megacolon;
- Known or suspected bowel obstruction;
- History of colorectal surgery.

### Bowel preparation regimen

Patients received elobixibat (Goofice^®^, EA Pharma Co., Ltd, Tokyo, Japan) 10 mg orally once daily for 3 consecutive days prior to colonoscopy. On the day of colonoscopy, bowel preparation was performed using OSS (Salprep^®^, Fuji Pharma Co., Ltd, Tokyo, Japan) according to the 1-day regimen (“one–two cup method”) recommended by the manufacturer. Salprep^®^ is supplied as a ready-to-drink solution in a 480 mL bottle. Bowel preparation was initiated on the morning of the examination day, approximately 6 hours before colonoscopy. In brief, OSS was administered on the morning of the examination day in a stepwise manner. One cup (approximately 180 mL) of OSS was ingested, followed by an additional cup of water (approximately 180–200 mL). This sequence was repeated (i.e. one cup of OSS followed by one cup of water) until adequate bowel evacuation was achieved. Stool adequacy was assessed based on predefined criteria, defined as the passage of clear or pale yellow liquid stool without solid residue. Patients were instructed to evaluate stool appearance during preparation, and OSS ingestion was discontinued once these criteria were met. Stool adequacy was primarily judged by the patients themselves, with additional guidance provided by medical staff when necessary. If adequate effluent was not achieved, additional OSS and water were administered according to the same alternating pattern. Colonoscopy was performed after completion of OSS ingestion, typically within a few hours of the last dose, according to routine clinical scheduling.

Regarding dietary instructions, patients were advised to avoid high-fiber foods on the day before colonoscopy and to refrain from eating after 9 p.m. However, no strict dietary restrictions (e.g. low-residue diet) were systematically enforced in this study.

### Endoscopic procedure

The colonoscopic examinations included in this study were conducted by six board-certified endoscopists with expertise in diagnostic and therapeutic colonoscopy. All endoscopic images were obtained using colonoscopes (PCF-H290AZI; Olympus Optical, Tokyo, Japan, or EC-L600ZP7; Fujifilm, Tokyo, Japan) with standard video processors (EVIS LUCERA system; Olympus, Tokyo, Japan, or LASEREO; Fujifilm, Tokyo, Japan). Bowel cleansing quality was evaluated under conventional white-light imaging.

### Outcome measures

The primary endpoint was the proportion of patients achieving adequate bowel preparation. Cleansing quality was assessed using the Boston Bowel Preparation Scale (BBPS) [10]. The colon was divided into three segments (right colon, transverse colon, and left colon), and each segment was scored from 0 to 3 according to the BBPS. Adequate overall bowel preparation was defined as a total BBPS score ≥6. In addition, adequate segmental cleansing was defined as a BBPS score ≥2 in each individual colonic segment. Secondary outcomes included adverse events, total OSS volume consumed, and patient-reported tolerability and acceptability.

Adverse events occurring during bowel preparation were prospectively recorded. These included nausea, vomiting, abdominal pain, dizziness, electrolyte abnormalities (if clinically suspected), and any other symptoms requiring medical attention. Although laboratory parameters, including serum electrolytes and renal function, were not systematically measured, electrolyte abnormalities were assessed only if clinically suspected. Serious adverse events were defined as events requiring hospitalization, emergency intervention, or procedure cancelation. During colonoscopy, patients were continuously monitored using standard parameters, including blood pressure, heart rate, oxygen saturation (SpO_2_), respiratory rate, and level of consciousness.

The total volume of OSS ingested by each patient was recorded. The mean volume was calculated, and the proportion of patients completing bowel preparation with ≤720 mL of OSS (approximately 1.5 bottles) was determined as an indicator of reduced-volume feasibility.

Patient perceptions were assessed using a structured self-administered questionnaire designed to evaluate the experience of bowel cleansing agents. The questionnaire consisted of the following domains: (i) Previous colonoscopy experience: patients were asked whether they had previously undergone colonoscopy (first time or second or more times). (ii) Palatability and ease of ingestion: patients evaluated the drinkability of the preparation, including taste and smell. Response options were: easy to drink or neutral or difficult to drink or unable to drink. (iii) Volume burden: patients were asked about the perceived amount of medication and fluid intake. Response options were: able to drink without difficulty or slightly large volume but manageable or too large to complete. (iv) Comparison with previous bowel preparations (for patients with prior colonoscopy experience). Response options were: previous preparation was more burdensome or current preparation was more burdensome or not applicable (first colonoscopy). (v) Preference for future examinations: patients were asked whether they would prefer the same preparation for future colonoscopy. Response options were: prefer the same preparation or prefer a different preparation.

### Statistical analysis

Given the exploratory nature of this single-arm pilot study, no formal sample size calculation was performed. The study was designed to evaluate feasibility, cleansing efficacy, safety, and tolerability in a real-world clinical setting. Continuous variables are presented as median (range) according to data distribution. Categorical variables are expressed as counts and percentages. The proportion of patients achieving adequate bowel preparation (total BBPS score ≥6) and the proportion of patients completing bowel preparation with ≤720 mL of OSS were calculated descriptively. Observed rates were compared descriptively with commonly reported adequate preparation rates in the literature; however, no formal comparative statistical testing was performed. Given the pilot nature of the study, analyses were primarily descriptive.

## Results

### Patient characteristics

Patient characteristics are summarized in Table 1. A total of 28 patients were included in the analysis. The median age was 58.5 years (range, 22–64 years). There were 17 men and 11 women. Twenty patients (71%) had a prior history of colonoscopy, while eight patients (29%) were undergoing colonoscopy for the first time. The indications for colonoscopy were positive fecal occult blood test in seven patients (25%), post-polypectomy surveillance in 14 patients (50%), constipation in one patient (4%), and other indications in six patients (21%). None of the patients were receiving anticoagulant therapy.

**Table 1:** Baseline clinical characteristics of the study population.

Characteristic	Patient (*n *= 28)
Age, years, median (range)	58.5 (22–64)
Male, *n* (%)	17 (61%)
Previous colonoscopy, *n* (%)	20 (71%)
Indication for colonoscopy, *n* (%)	
Positive fecal occult blood test	7 (25%)
Post-polypectomy surveillance	14 (50%)
Constipation	1 (4%)
Others	6 (21%)
Anticoagulant use, *n* (%)	0 (0%)

### Bowel cleansing efficacy

Detailed BBPS results for each colonic segment, including score distribution and overall adequacy rates, are presented in Table 2. Adequate bowel preparation, defined as a total BBPS score ≥ 6, was achieved in 27 of 28 patients (96%). This adequacy rate exceeds the commonly recommended benchmark of ≥90% for adequate bowel preparation in quality colonoscopy practice [1]. In addition, adequate segmental cleansing (defined as BBPS ≥ 2 in all three colonic segments) was achieved in 23 of 28 patients (82%). Segmental analysis showed that a BBPS score ≥ 2 was obtained in 23 of 28 patients (82%) in the right colon, 27 of 28 (96%) patients in the transverse colon, and 100% in the left colon. No procedure was canceled due to inadequate cleansing.

**Table 2:** Bowel cleansing quality assessed by the BBPS.

Variable	Patient (*n *= 28)
Adequate bowel preparation, *n* (%)	27 (96%)
Colon segment	
Right colon, *n* (%)	
BBPS 3	15 (53%)
BBPS 2	8 (29%)
BBPS 1	5 (18%)
BBPS 0	0 (0%)
Transverse colon, *n* (%)	
BBPS 3	19 (67%)
BBPS 2	8 (29%)
BBPS 1	1 (4%)
BBPS 0	0 (0%)
Left colon, *n* (%)	
BBPS 3	21 (75%)
BBPS 2	7 (25%)
BBPS 1	0 (0%)
BBPS 0	0 (0%)

BBPS = Boston Bowel Preparation Scale.

### Safety

Adverse events were minimal. Mild nausea occurred in one patient (4%). No serious adverse events were observed.

### OSS volume

The mean total OSS volume consumed was 771 mL. Sixteen patients (57%) completed bowel preparation with ≤720 mL of OSS.

### Tolerability

Patient-reported tolerability outcomes are summarized in Table 3. With respect to palatability, 23 patients (82%) reported that the preparation was easy to drink or neutral in taste. Regarding volume burden, all patients (100%) responded that the amount of medication and fluid was either able to be consumed without difficulty or slightly large but manageable. Twenty patients had prior colonoscopy experience. Among them, 19 had previously received PEG plus ascorbic acid solution, and one had received magnesium citrate as a bowel preparation agent. Among these patients, 15 (75%) reported that the current preparation was more tolerable compared with their previous bowel cleansing regimen. Furthermore, 24 patients (86%) expressed a preference for using OSS again in future colonoscopy examinations.

**Table 3:** Patient-reported tolerability and acceptability of bowel preparation.

Item		*n* (%)
Previous colonoscopy experience (*n *= 28)		
First time		8 (29%)
Second or more times		20 (71%)
Palatability and ease of ingestion (*n *= 28)		
Easy to drink		15 (53%)
Neutral		8 (29%)
Difficult to drink		5 (18%)
Unable to drink		0 (0%)
Volume burden (*n *= 28)		
Able to drink without difficulty		16 (57%)
Slightly large volume but manageable		12 (43%)
Too large to complete		0 (0%)
Comparison with previous preparation (*n *= 20)		
Previous preparation was more burdensome		15 (75%)
Current preparation was more burdensome		5 (25%)
Preference for future examinations (*n *= 28)		
Prefer the same preparation		24 (86%)
Prefer a different preparation		4 (14%)

## Discussion

Given the limited evidence regarding the combined use of elobixibat with OSS for colonoscopy preparation, we conducted this pilot study to explore feasibility before designing larger comparative trials. In this single-center pilot study, a bowel preparation regimen combining elobixibat with OSS demonstrated favorable feasibility, cleansing efficacy, safety, and patient tolerability in a real-world clinical setting. Overall, bowel preparation quality was acceptable, and no serious adverse events were observed.

Cleansing quality was assessed using the BBPS, one of the most widely validated and internationally accepted scoring systems. The BBPS evaluates bowel cleanliness after washing and suctioning during withdrawal and assigns a score from 0 to 3 to each of three colonic segments (right, transverse, and left colon), thereby reflecting the final mucosal visibility relevant to lesion detection [10]. Using a total BBPS score ≥6 as an indicator of good bowel preparation, 27 of 28 patients (96%) achieved adequate cleansing in our cohort. Segmental analysis further demonstrated that a BBPS score ≥2 was obtained in 82% of patients in the right colon, 96% in the transverse colon, and 100% in the left colon (Fig. 1). These findings suggest that this regimen achieved generally favorable bowel cleansing across colonic segments, although the right colon remained the most challenging area, consistent with prior observations in clinical practice and previous validation studies [11]. Previous investigations have shown that segments with BBPS scores ≥2 are associated with adequate mucosal visualization and clinically appropriate surveillance recommendations [11]. Conversely, lower segmental scores, particularly in the right colon, have been linked to higher miss rates for adenomas. Therefore, segment-level analysis is clinically meaningful and strengthens the interpretation of cleansing efficacy beyond total scores alone. OSS has been shown in randomized trials to provide cleansing efficacy comparable to PEG-based regimens with lower volume intake [5, 6]. While our study was not designed as a comparative trial, the high overall adequacy rate and favorable segmental scores observed in this feasibility setting support the practical applicability of this regimen. Nonetheless, because this was a single-arm pilot study, causal inference regarding the contribution of individual regimen components cannot be made, and further comparative studies are warranted to validate these findings.

Safety is a critical consideration when combining pharmacologic agents for bowel preparation. OSS is a hyperosmotic preparation that induces catharsis through osmotic fluid shifts within the intestinal lumen. Randomized controlled trials have demonstrated that OSS has a safety profile comparable to PEG-based regimens, with most adverse events being mild and transient gastrointestinal symptoms such as nausea or abdominal discomfort [5, 6]. Clinically significant electrolyte disturbances or renal impairment are uncommon in appropriately selected patients without pre-existing risk factors [12]. Elobixibat, an ileal bile acid transporter inhibitor, promotes colonic motility by increasing bile acid delivery to the colon and enhancing fluid secretion. Phase 3 trials in patients with chronic constipation have demonstrated an acceptable safety profile, with diarrhea and abdominal pain being the most frequently reported adverse events, while serious adverse events are rare [7, 13]. Because elobixibat acts locally in the gastrointestinal tract with minimal systemic absorption, systemic toxicity is considered unlikely [14]. Theoretically, combining OSS with elobixibat could potentiate fluid shifts or increase the risk of dehydration or electrolyte imbalance due to additive cathartic effects. However, no such safety signals were observed in the present study. Only one patient experienced mild nausea, and no serious adverse events occurred. Importantly, we excluded patients with significant renal dysfunction, cardiovascular instability, or conditions predisposing to fluid-electrolyte disturbances, in accordance with current guideline recommendations emphasizing careful patient selection for bowel preparation agents [1, 2]. Previous studies evaluating bowel preparation safety underscore that patient comorbidities, rather than the preparation agent alone, are major determinants of adverse outcomes [12, 15]. In this context, the absence of serious adverse events in our cohort suggests that the OSS plus elobixibat regimen is safe in a relatively low-risk, non-elderly population.

**Figure 2:**
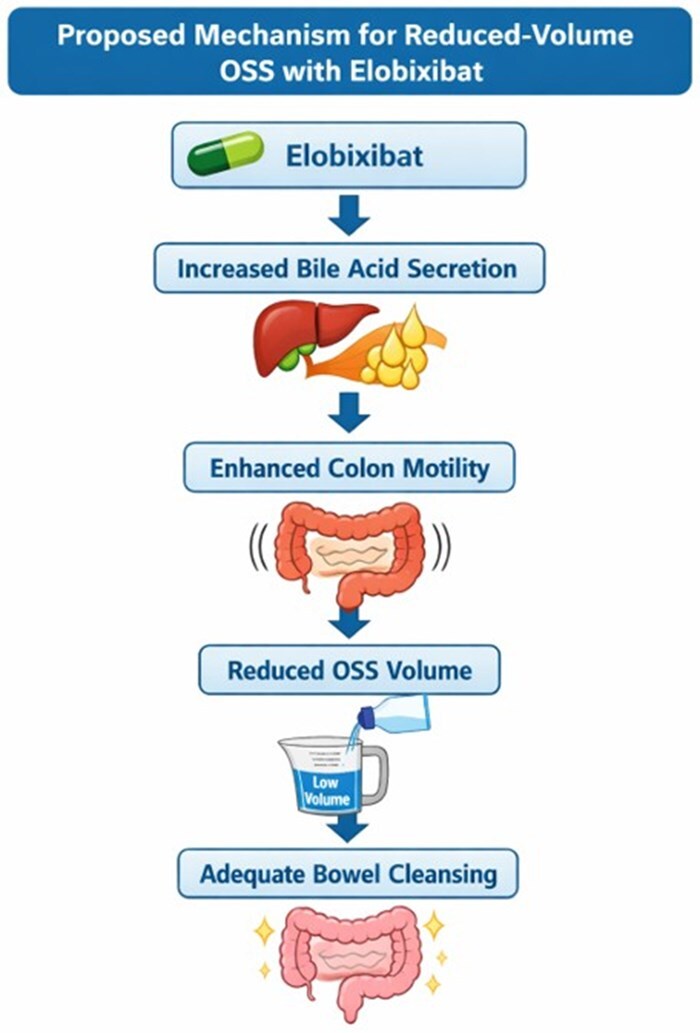
Graphical summary of the proposed mechanism of reduced-volume bowel preparation using elobixibat combined with oral sulfate solution (OSS). Elobixibat inhibits the ileal bile acid transporter, resulting in increased delivery of bile acids to the colon. This mechanism enhances colonic secretion and motility, thereby facilitating bowel evacuation. As a result, adequate bowel cleansing may be achieved with a reduced volume of oral sulfate solution in selected patients.

Reducing the volume of bowel preparation is clinically meaningful because ingestion burden is a major determinant of adherence and overall patient experience. Randomized trials and meta-analyses have shown that low-volume, split-dose regimens can achieve cleansing efficacy comparable to high-volume preparations while improving tolerability and completion rates [3, 4]. However, in most previous studies, volume reduction has been achieved through split-dose administration, in which intake is divided over two time periods. In contrast, the present study employed a same-day (1 day) regimen rather than a split-dose protocol, yet a substantial proportion of patients completed bowel preparation with a reduced OSS volume. In Japan, the standard recommended regimen for Salprep^®^ consists of two 480 mL bottles (total 960 mL) administered according to the 1-day protocol. In the present study, the mean total OSS intake was 771 mL, and 57% of patients achieved adequate bowel preparation with ≤720 mL using a tailored “stop-when-clear” strategy. Thus, many patients were able to complete preparation with a volume lower than the standard recommended amount. These findings suggest that volume reduction may be feasible even within a same-day framework, and that the required OSS volume may not need to be fixed for all patients in carefully selected populations. OSS was originally developed as a reduced-volume alternative to conventional high-volume lavage solutions, and randomized trials have supported its efficacy and safety compared with standard 4 L preparations [5, 6, 16]. A pilot study in Japanese patients further suggested that reduced-volume OSS-based preparation is feasible and safe in clinical practice [17]. Nevertheless, most prior low-volume strategies have relied on split-dose approaches [3, 4], making the present findings notable in demonstrating potential volume reduction within a 1-day administration regimen. A plausible mechanistic explanation for this observation is the adjunctive use of elobixibat. By inhibiting the ileal bile acid transporter, elobixibat increases colonic bile acid delivery, thereby enhancing secretion and motility [7, 8]. The administration of elobixibat for three consecutive days prior to colonoscopy may have further contributed to this effect by allowing sustained bile acid accumulation in the colon, resulting in more consistent stimulation of colonic motility and secretion at the time of bowel preparation. The proposed physiological mechanism by which elobixibat may facilitate bowel cleansing and potentially reduce the required volume of oral sulfate solution is illustrated in Fig. 2. Clinical studies have shown that elobixibat can facilitate bowel cleansing when incorporated into low-volume regimens, including a prospective randomized multicenter trial in which elobixibat combined with 1 L PEG-ascorbate achieved cleansing efficacy comparable to a commonly used comparator regimen [9]. Although those studies used PEG-based preparations rather than OSS, they support the concept that elobixibat may augment cathartic efficacy and potentially allow further reduction in lavage volume.

It is also important to consider differences in OSS formulation between Japan and European and North American countries when interpreting volume-related outcomes. In Japan, Salprep^®^ (Fuji Pharma Co., Ltd, Tokyo, Japan) is supplied as a ready-to-drink solution in a 480 mL bottle and is administered with additional water according to a 1-day protocol. In contrast, in North America, SUPREP^®^ (Braintree Laboratories, Inc., Braintree, MA, USA) is provided as a smaller-volume concentrate (approximately 176–177 mL per dose) that is diluted with water to approximately 473 mL before ingestion, followed by additional water intake. The standard regimen consists of two doses (typically administered as a split dose). Similarly, in European countries, a sulfate-based concentrate formulation is marketed as Eziclen^®^ (Ipsen Pharma, Paris, France), in which each 176 mL bottle is diluted with water to approximately 500 mL prior to administration, and two doses are generally recommended. Although these products share sulfate-based osmotic components, their formulation designs, total administered volumes, and dosing protocols differ substantially. Such differences may influence perceived volume burden, drinking experience, and flexibility of dose adjustment.

Furthermore, patient-reported outcomes are increasingly recognized as important determinants of colonoscopy quality, as preparation-related discomfort can negatively affect adherence to screening and surveillance programs. In the present study, 82% of patients reported that the preparation was easy to drink or neutral in palatability, and all patients indicated that the volume was either manageable or only slightly burdensome. Furthermore, among patients with previous colonoscopy experience, 75% reported that the current regimen was more tolerable than their prior preparation, and 86% expressed a preference to use the same preparation for future examinations. The palatability of bowel preparation agents has been shown to significantly influence patient compliance and completion rates. Studies comparing low-volume preparations with conventional high-volume PEG regimens have consistently demonstrated improved patient satisfaction and willingness to repeat the same regimen when volume burden is reduced [3, 18]. Poor taste and excessive volume are frequently cited as the most distressing aspects of bowel preparation [19]. Therefore, the favorable responses regarding drinkability and volume burden observed in our cohort are clinically relevant. In addition, willingness to reuse the same preparation for future colonoscopy is a meaningful patient-centered outcome. Previous research has shown that patient preference strongly correlates with adherence to repeat colonoscopy recommendations, which is particularly important in surveillance settings [20]. The high proportion of patients in our study who expressed a desire to use the same preparation again suggests that this regimen may support long-term adherence to colonoscopic screening or surveillance strategies.

This study has several limitations. First, it was a single-center, single-arm pilot study with a small sample size and without a control group, which limits causal inference regarding the incremental effect of elobixibat or the independent contribution of reduced OSS volume. Selection bias cannot be excluded, as enrollment was limited to a specific institutional setting. Second, the study population consisted of relatively low-risk, non-elderly patients without significant renal or cardiovascular comorbidities. Although this approach was appropriate for safety considerations, it restricts generalizability to elderly patients and those with higher clinical risk profiles, who represent an important proportion of individuals undergoing colonoscopy [1, 15]. The extensive exclusion criteria, including age ≥65 years, renal dysfunction, and the use of medications affecting renal blood flow, resulted in a highly selected population that may not reflect routine clinical practice. Therefore, caution is warranted when extrapolating these findings to broader, real-world populations. Third, bowel cleansing quality was assessed using the validated BBPS [10, 11]; however, interobserver variability was not formally evaluated, and assessment was performed by the endoscopists conducting the procedures, which may introduce observational bias. Moreover, no formal calibration or training session was conducted among participating endoscopists, which may have contributed to variability in scoring. Fourth, patient-reported tolerability was assessed using a structured but non-validated questionnaire. Although clinically relevant domains were captured, measurement bias and recall bias cannot be excluded, particularly in patients with prior colonoscopy experience, where comparisons with previous preparations were based on subjective retrospective assessment. Finally, laboratory parameters, such as electrolyte levels, were not systematically measured before and after preparation. Objective measures of hydration status were also not assessed, and safety evaluation relied primarily on clinical observation. Therefore, subclinical electrolyte disturbances, such as hyponatremia or hypokalemia, cannot be excluded, particularly given the osmotic mechanism of OSS and the potential additive effects of elobixibat on fluid and electrolyte balance [12].

## Conclusion

In this single-center pilot study, a same-day bowel preparation regimen combining elobixibat with OSS demonstrated favorable feasibility, acceptable cleansing efficacy, and good patient tolerability in a selected non-elderly population. Adequate bowel preparation was achieved in most patients, and no serious adverse events were observed. Notably, more than half of the patients completed preparation with ≤720 mL of OSS, suggesting that individualized volume reduction may be achievable within a 1-day administration framework. Further well-designed comparative studies are warranted to clarify the incremental contribution of elobixibat and to determine the optimal minimal OSS volume for broader patient populations.
